# Atomic scale interfacial magnetism and origin of metal-insulator transition in (LaNiO$_3$)$_n$/(CaMnO$_3$)$_m$ superlattices: a first principles study

**DOI:** 10.1038/s41598-023-30686-w

**Published:** 2023-03-28

**Authors:** J. Jilili, I. Tolbatov, F. Cossu, A. Rahaman, B. Fiser, M. Upadhyay. Kahaly

**Affiliations:** 1 ELI ALPS, ELI-HU Non-Profit Ltd., Wolfgang Sandner utca 3., Szeged, H-6728 Hungary; 2 Department of Pharmacy, University of Chieti-Pescara “G. d’Annunzio”, Chieti, Italy; 3 Asia Pacific Center for Theoretical Physics, Pohang, 37673 Korea; 4 School of Mechanical Engineering, Vellore Institute of Technology, Vellore, 632014 India; 5 Higher Education and Industrial Cooperation Centre, University of Miskolc, Miskolc, 3515 Hungary; 6 Department of Physical Chemistry, University of Lodz, 90-236 Lodz, Poland; 7 Ferenc Rakoczi II Transcarpathian Hungarian College of Higher Education, 90200 Beregszász, Ukraine; 8 Department of Physics and Institute of Quantum Convergence, Kangwon National University, 24341 Chuncheon, Korea; 9 Institute of Chemical Research of Catalonia (ICIQ), The Barcelona Institute of Science and Technology, Av. Paisos Catalans 16, 43007 Tarragona, Spain

**Keywords:** Materials science, Nanoscience and technology, Physics

## Abstract

Interfacial magnetism and metal-insulator transition at LaNiO$_3$-based oxide interfaces have triggered intense research efforts, because of the possible implications in future heterostructure device design and engineering. Experimental observation lack in some points a support from an atomistic view. In an effort to fill such gap, we hereby investigate the structural, electronic, and magnetic properties of (LaNiO$_3$)$_n$/(CaMnO$_3$)$_m$ superlattices with varying LaNiO$_3$ thickness (*n*) using density functional theory including a Hubbard-type effective on-site Coulomb term. We successfully capture and explain the metal-insulator transition and interfacial magnetic properties, such as magnetic alignments and induced Ni magnetic moments which were recently observed experimentally in nickelate-based heterostructures. In the superlattices modeled in our study, an insulating state is found for *n*=1 and a metallic character for *n*=2, 4, with major contribution from Ni and Mn 3*d* states. The insulating character originates from the disorder effect induced by sudden environment change for the octahedra at the interface, and associated to localized electronic states; on the other hand, for larger *n*, less localized interfacial states and increased polarity of the LaNiO$_3$ layers contribute to metallicity. We discuss how the interplay between double and super-exchange interaction via complex structural and charge redistributions results in interfacial magnetism. While (LaNiO$_3$)$_n$/(CaMnO$_3$)$_m$ superlattices are chosen as prototype and for their experimental feasibility, our approach is generally applicable to understand the intricate roles of interfacial states and exchange mechanism between magnetic ions towards the overall response of a magnetic interface or superlattice.

## Introduction

Perovskites are among the most popular functional materials in the research fields of condensed matter physics and material science and often used for applications in nanotechnology^1, 2^. Similarity in their chemical composition and ionic coordination (with few differences) has contributed to a huge understanding of their general properties from a theoretical perspective. In addition, efficient growth techniques for perovskite oxides, such as radio frequency magnetron sputtering^3^, pulsed laser deposition^4^ and molecular beam epitaxy^5^ have enhanced the popularity of this class of materials in the development of applications in energy storage/harvesting and information field, creating significant research interest^6–8^. This brings in wealth of possibilities in new design and modification of perovskite interfaces as well, a primary tool to harness the full potential of this class of materials, and for accessing a wide range of functionalities as well as novel ground states at the interface.

Lack of stoichiometry and/or inversion symmetry at surfaces or interafces, cation intermixing, doping, epitaxial strain or applied pressure can induce or tune magnetism at the interface, with a possible coupling between these localized defects and the magnetic state. For example, interfacial magnetism in LaMnO$_3$/SrMnO$_3$^9^, CaRuO$_3$/CaMnO$_3$^10^ superlattices, BiMnO$_3$/SrTiO$_3$^11^ and LaAlO$_3$/SrTiO$_3$ superlattices^12^, two-dimensional electron gas in LaAlO$_3$/SrTiO$_3$^13^, metallicity in LaTiO$_3$/SrTiO$_3$^14^, exchange bias in (111)-oriented LaNiO$_3$/LaMnO$_3$^15^, polar mismatch in LaNiO$_3$/CaMnO$_3$ superlattices^16^, orbital order in LaMnO$_3$/SrMnO$_3$ superlattices^17^, have recently been found. Although these examples may suggest a major role of the electronic degrees of freedom in the occurrence of the above mentioned exotic phenomena, research in the last decade has widely demonstrated that a thorough analysis must be carried out to understand their origins (see, for example, the case of LaAlO$_3$/SrTiO$_3$ interface, and a recent analysis by Yu and Zunger^18^).

In fact, as competing phases get closer, subtle differences (in the strain and/or stoichiometry) may induce dramatic changes – such as metal-insulator or magnetic order transitions and even the presence of superconducting regimes. Jahn-Teller modes combined with octahedral rotations and their connectivity (i.e. the rotations and tiltings of octahedra relative to one another) play an important role in interfacial structure and property control in short-period superlattices. In this regard, Glazer^19^ first developed a systematic description of the possible tilting patterns in perovskites using group-theoretical analysis in 1972; later on, in 1998, Stokes *et al*^20^ classified that there are 15 possible tilting patterns in perovskites. The role of the octahedral network on the emergence of novel magnetic properties of the perovskite heterostructures has been highlighted recently^21, 22^, elucidating the intricacies of the connection and suggesting possible research routes to achieve desired properties in engineered interfaces.

In this respect, perovskites in lower dimensional environments (such as surfaces or interfaces) host phases in competition which may be elusive though. For example, charge vs orbital order, as predicted recently^15, 23–25^ are difficult to detect, possibly because of the peculiarity of samples. Control and detection of defects is not trivial, and even the detailed formalism of density functional theory (DFT), which has achieved many predictions (e.g. the ferromagnetic ground state in LaMnO$_3$/SrTiO$_3$ interfaces^12^) needs powerful tools to overcome the complexity of electronic correlation; an important part of the experimental work and analysis relies on DFT to best interpret their results. Suzuki and coworkers have observed interfacial ferromagnetism (FM) in LaNiO$_3$/CaMnO$_3$ superlattices, despite their constituents are not ferromagnetic^26^. Pristine CaMnO$_3$ is a G-type antiferromagnetic (AFM) insulator, which was used for applications in spintronics since the discovery of colossal magneto-resistance in 1990s^27^. Recently, the role of surface relaxation on the magnetic properties has been identified as a crucial aspect^28^; in fact, the bulk G-type AFM order (where all nearest neighbors have an AFM alignment) is maintained at the surface^29^, despite a pure electronic reconstruction tends to favour a FM coupling^30^. The effect of LaNiO$_3$ at the interface demonstrates the importance of octahedra rotations and connectivity^31^ in determining the electronic and magnetic properties; the interfacial FM was further ascribed also to polarity-mismatch-driven charge transfer and exchange interaction^16^. A relation with the LaNiO$_3$ layer thickness^26^ was also found. On the other hand, LaNiO$_3$ is paramagnetic metallic in the bulk, but thin films and superlattices of LaNiO$_3$ show significantly different properties, such as metal-insulator transition^32^, increased conductivity^33^, strain induced phase transition^34^. Octahedral connectivity determines the electronic and magnetic properties also in nickelate/manganite superlattices, as (111)-oriented superlattices ones^15, 35^ have dramatically different properties than the (001)-oriented ones^35, 36^.

Despite wide research exists on CaMnO$_3$ and LaNiO$_3$ in bulk, surface and interfaces, and that synthesis and experimental analysis of CaMnO$_3$/LaNiO$_3$ superlattices are available since a few years, theoretical investigation of LaNiO$_3$/CaMnO$_3$ superlattices is not reported yet to the best to our knowledge. We believe that delving into this system contributes to the understanding of magnetic perovskite interfaces, also in view of technological applications. Therefore, we present an *ab-initio* study of the structural, electronic, and magnetic properties of the LaNiO$_3$/CaMnO$_3$ superlattices with 1:8, 2:8 and 4:8 thickness ratios, clarifying the possible origin of the induced magnetism and phase transition. On the basis on the evolution of the octahedra distortion, electronic properties and interfacial magnetism in the superlattices, we discuss how the structural change correlates with corresponding charge transfer and magnetism for various LaNiO$_3$ thicknesses. Our results have implications for interpretation of the experimentally observed^16^ magnetism and metal-insulator transition in similar systems^37, 38^.

## Computational details

We performed DFT calculations with the Vienna Ab-initio Simulation Package^39–41^ and employed the generalized gradient approximation in the parametrization of Perdew, Burke, and Ernzerhof^42^. We employ the pseudopotentials generated with the projector augmented-wave method^43, 44^ with 10, 11, 13, 10 and 6 valence electrons for Ca, La, Mn, Ni and O. The total energy cutoff is 400 eV and the energy tolerance is 10$^{-5}$ eV for the electronic loop. Structures are considered relaxed when the forces are smaller than (0.01eV/Å ). Due to the correlated nature of the localized 3*d* orbitals of Ni and Mn, a Hubbard correction in the scheme of Dudarev^45^ is used, with $U_{eff}$ = 4.3 eV and $U_{eff}$ = 3.0 eV on Ni and Mn, respectively, in agreement with previous works^46, 47^.

Since we aim at modeling the LaNiO$_3$ and CaMnO$_3$ superlattice as if they are epitaxially grown on a LaAlO$_3$ substrate, we set the in-plane lattice constants of all superlattices to that of the substrate (LaAlO$_3$:3.789Å)^48–50^. The (LaNiO$_3$)$_n$/(CaMnO$_3$)$_m$ superlattices are modeled with a $\sqrt{2}$ $\times$ $\sqrt{2}$ in plane supercell of the pseudo-cubic perovskite structure, which is sufficient to take into account the possible magnetic orderings as well as the tilts and rotations of the octahedra. We select *n*=1, 2, 4 by considering the phase transition threshold thickness range of LaNiO$_3$^33, 51^, while *m* is set to 8 in all cases (schematic view in Fig. 2). The out-of-plane lattice constants for superlattices are taken from the weighted averaged optimized bulk values.

In agreement with the construction of the supercells, we sample the reciprocal space with a Monkhorst-Pack scheme for the generation of the reciprocal points, with *k*-meshes of $12\times ~12\times ~2$ for all superlattices. Due to the superlattice construction, there are two types of interfaces; we denote them as *n*-type (La$^{3+}$O$^{2-}$)$^+$/(Mn$^{4+}$O$^{2-}_2$)$^0$ interface (since ideally there is one extra electron) and *p*-type (Ca$^{2+}$O$^{2-}$)$^0$/(Ni$^{3+}$O$^{2-}_2$)$^-$ interface, where they refer to the compensating charges formed at the corresponding interfaces.

## Results and discussion

### Structural properties

The schematic view of the optimized structures of 1:8, 2:8 and 4:8 superlattices are shown in Fig.2. With the in-plane lattice constant set to the value of LaAlO$_3$, the out-of-plance lattice constants for the superlattices are optimized, taking the values of 3.94 Å and 3.73 Å per unit cell, for the LaNiO$_3$ and CaMnO$_3$, respectively. With respect to its bulk value of 1.99 Å, the Ni-O bond length at the *p*-IF decreases by 0.04 Å to 0.07 Å with increasing LaNiO$_3$ thicknesses due to the possibility of formation of localized interface bonds, consistent with previous studies^52, 53^, see our results in Table 1. Note the prominently larger values of Ni-O bond lengths at the *n*-IF as compared to the *p*-IF, due to the exposure of the NiO$_2$ layers to different environment such as NiO$_2$-LaO-MnO$_2$(*n*-IF) or NiO$_2$-CaO-MnO$_2$(*p*-IF). Depending on the growth sequence, such intrinsic interfacial structural asymmetry were previously obtained also in LaMnO$_3$/LaNiO$_3$ heterostructures^54^. While the former yields longer bond length since half of the cell belongs to LaNiO$_3$, the latter yields shorter bond length since half of the cell belongs to CaMnO$_3$. In CaMnO$_3$, along the *z* axis, we observe an off-centering of Mn from the mid-point of the MnO$_6$ octahedra towards the *p*-IF by up to 0.05 Å, which implies a possible induced polarity in the CaMnO$_3$ region of the superlattices. This suggests a correlation between strain and polarity, in agreement with tensile-strain-induced polar distortions reported elsewhere^55^. However, such effect reduces dramatically in the 1:8 system possibly due to a reduced polar effect from LaNiO$_3$ (due to a reduced thickness).

**Table 1:** Interfacial bond lengths($l_B$), bond angles($\theta$) along *z* axis and magnetic moments (*m*).

		1:8		2:8		4:8	
		*n* interface	*p* interface	*n* interface	*p* interface	*n* interface	*p* interface
$l_B$(Å)	Mn-O	1.90	1.89	1.92	1.91	1.93	1.90
$l_B$(Å)	Ni-O	2.39	1.95	2.27	1.93	2.23	1.92
$\theta$	O-Mn-O	174.4$^{\circ }$	178.4$^{\circ }$	176.1$^{\circ }$	178.7$^{\circ }$	175.6$^{\circ }$	178.4$^{\circ }$
$\theta$	O-Ni-O	165.5$^{\circ }$	165.5$^{\circ }$	179.8$^{\circ }$	174.7$^{\circ }$	178$^{\circ }$	174$^{\circ }$
$\theta$	Ni-O-Mn	170.1$^{\circ }$	159.5$^{\circ }$	163.0$^{\circ }$	154.0$^{\circ }$	163.4$^{\circ }$	153.5$^{\circ }$
*m* ($\mu _B$)	Ni	1.195/1.193	1.195/1.193	−1.347/−1.358	0.894/0.883	1.331/1.326	0.807/0.814
*m* ($\mu _B$)	Mn	3.017/3.017	3.063/3.061	3.013/3.013	3.047/3.047	3.067/3.067	3.030/3.030

The interfacial octahedral rotation pattern in a superlattice is the combined effect of the strain (introduced by slight lattice mismatch of the substrate and the film) and the octahedral connectivity of different components. From the optimized structures, we observe that both octahedra types (NiO$_6$ and MnO$_6$) maintain the a$^{-}$a$^{-}$c$^{+}$ tilt pattern of the manganite in the bulk. The a$^{-}$a$^{-}$c$^{+}$ tilt pattern consists of out-of-phase rotations of equal magnitude about two Cartesian axes along (*x*, *y*), while exhibiting different magnitude of in-phase-rotations (positive sign as superscript) along the third orthogonal direction^55^. We find this angular distortion for the connecting Ni-O-Mn bond angles to be prominent, see Table 1 for the 2:8 and 4:8 cases, while for the 1:8 case it is less pronounced. This shows substantially suppressed tilt pattern in LaNiO$_3$ region with clear reduction of in plane rotations.

The relaxed structures show that layers with odd and even numbers of unit cells of LaNiO$_3$ have a different bonding character, suggesting a frustration of octahedra tilts^56^. Valence states of magnetic cations, M-O-M (M = Mn, Ni) bond angles and M-O bond length are key factors on determining the transport properties of the superlattice. The Ni-O-Mn bond angles at the *n*-IF are up to 10$^{\circ }$ larger than those at the *p*-IF (see Table 1) which resembles the bulk LaNiO$_3$ (165.2$^{\circ }$^57^) and CaMnO$_3$ (157.7$^{\circ }$^58^) bond angles. This is due to the fact that the O ligands at the *n*-IF(*p*-IF) are surrounded by La (Ca) cations, and therefore influenced more by the nickelate (manganite) environment.

However, the octahedral connectivity at 1:8 case is substantially different from other cases; for instance, up to 7$^{\circ }$ larger Ni-O-Mn angles (for both *n*-IF and *p*-IF) are obtained due to strong orbital and charge redistribution. This difference in octahedra connectivity affects the change in densities of states at and around the Fermi level (E$_F$). The average Mn–O–Mn bond angles along *z* axis is 156$^{\circ }$ while Ni–O–Ni angle is 163.5$^{\circ }$ which indicates more tilted MnO$_6$ octahedra pattern than NiO$_6$ octahedra. The NiO$_6$ octahedra shortens up to 0.1 Å along *z* axis at *p*-IF in 2:8, 4:8 cases whereas it elongates up to 0.35 Å at *n*-IF. In the 1:8 case NiO$_6$ shows only elongation along the *z* axis. This is due to the fact that the MnO$_6$(NiO$_6$) octahedra directly expose to the *n*-IF(*p*-IF) (MnO$_6$ (NiO$_6$) octahedra are the main components of the n-IF (p-IF)), therefore, more prominent structural redistribution takes place at corresponding interfaces. Note that Ni-O-Mn bond angles decrease with increasing LaNiO$_3$ thickness at *p*-IF which hints the possible weakening of the exchange interaction between magnetic atoms. Resulting modification of density of electronic states of these superlattices are discussed in the next section.

### Electronic properties

**Figure 1:**
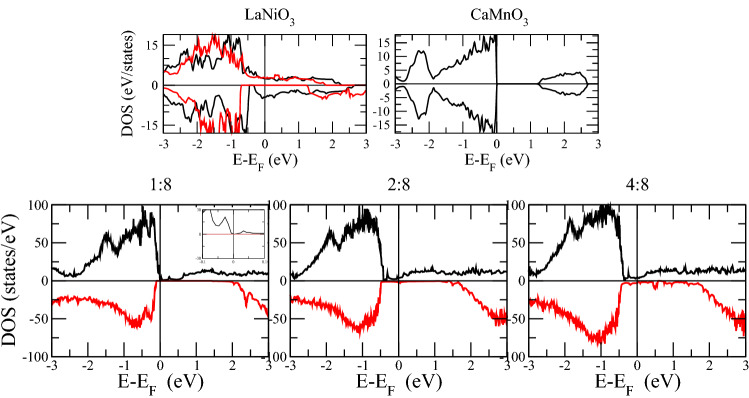
Total densities of states of strained bulks LaNiO$_3$ and CaMnO$_3$ and superlattice LaNiO$_3$/CaMnO$_3$ with varying LaNiO$_3$ thicknesses.

Before delving into the properties of the superlattices, we note that the insulating character of AFM ordered CaMnO$_3$ is well reproduced with $U_{eff}$=3 eV on Mn 3*d* states, see Fig.1. Without the inclusion of $U_{eff}$, the LaNiO$_3$ system shows metallic character whereas with $U_{eff}$=4.3 eV^46^, the DOS shows half-metallic character which is similar to earlier study^59^.

**Figure 2:**
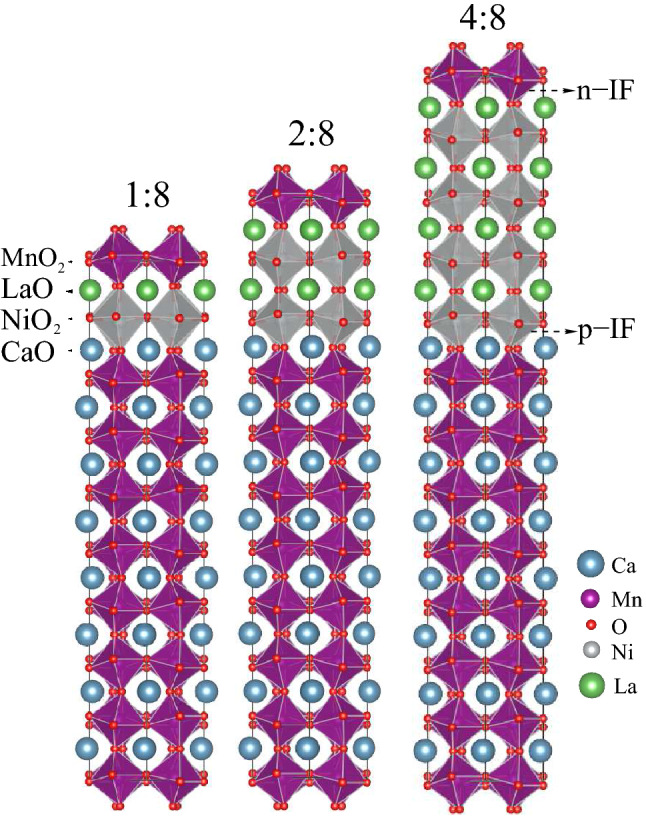
The schematic view of the optimized (LaNiO$_3$)$_n$/(CaMnO$_3$)$_m$ superlattices where *n*:*m* = 1:8, 2:8 and 4:8. The purple and gray octahedra represent the MnO$_6$ and NiO$_6$, respectively.

With reference to Fig.1, showing the total DOS, we analyze the overall electronic character of the superlattices. The 1:8 superlattice shows insulating character, with the valence states of the majority spin channel dropping to zero just below E$_F$, see inset of Fig.1, and the minority spin channel exhibiting a gap as large as 1.5 eV. Both channels have a dramatic drop of states below E$_F$ – the drop in the minority spin channel occurs at slightly lower energy. Moving to a larger LaNiO$_3$ thickness, we notice that the DOS of both spin channels drop at lower energies as compared to 1:8 thickness, with relative (and mostly rigid) shift of the DOS peaks towards lower energy. However, they exhibit non negligible states at E$_F$ (for both spin channels). The 4:8 superlattice shows an enhancement of the metallic character but with no qualitative difference with respect to the 2:8 case.

**Figure 3:**
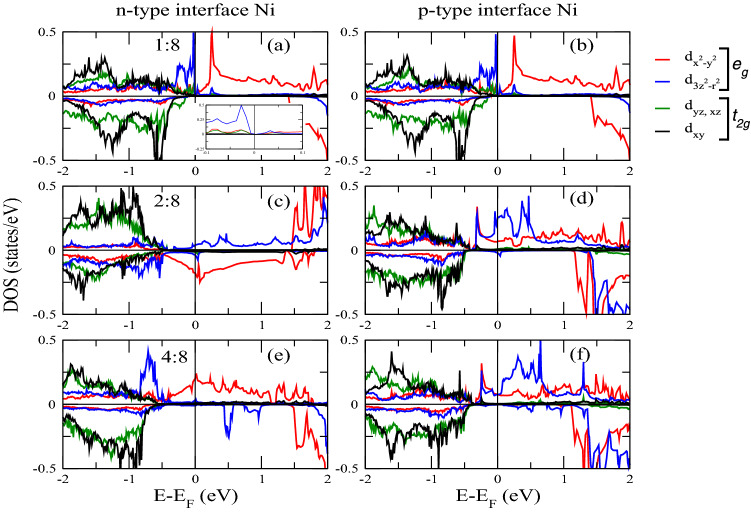
Projected densities of states of Ni atoms.

**Figure 4:**
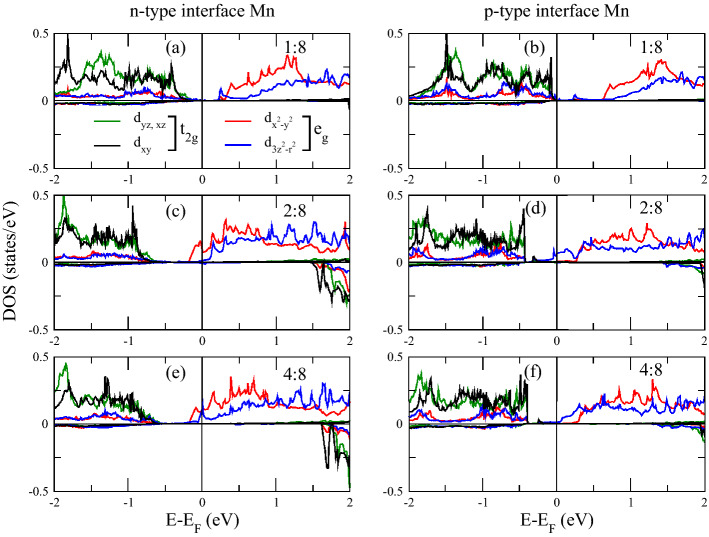
Projected densities of states of Mn atoms.

We now analyze the projected DOS of Ni and Mn atoms at respective interfaces, see in Figs.3 and 4. The electronic contribution from the $e_{g}$ (formed of $d_{x^{2}-y^{2}}$ and $d_{3z^2-r^{2}}$ ) sub-orbitals is prominent near E$_F$ compared to the $t_{2g}$’s ($d_{xy}$, $d_{xz}$ and $d_{yz}$). Obviously, the Ni layer is both at the *n*-IF and at the *p*-IF in the 1:8 superlattice. Analyzing the Mn DOS for all superlattices, we can ascribe the sudden drop in the total DOS of all superlattices below E$_F$ to the $t_{2g}$’s, because the trend of the total and local Mn DOS – Figs.1 and 4 respectively – coincide for all cases. In the 1:8 case, such trend is accompanied by the Ni $d_{3z^2-r^{2}}$ which contributes alone to the DOS around E$_F$, unlike the other cases where, the $e_{g}$’s have a balanced contribution. In other words, the Ni $e_{g}$ DOS is pinned by the Mn DOS at the interface in the 1:8 case, providing a signature for electron localization which is removed for thicker LNO layers.

The usual orbital ordering holds for the 2:8 and 4:8 superlattices, while the spectral weight of the Ni $e_{g}$ orbitals stays around E$_F$ at the *p*-IF – where half of the Ni crystal field is governed by the manganite – its contribution at E$_F$ is smaller at the *n*-IF. Furthermore, the contribution at E$_F$ to the $d_{x^{2}-y^{2}}$ Ni DOS has opposite magnetization with respect to the $d_{3z^2-r^{2}}$, whereas at *p*-IF both states contribute to majority spin channel, see Fig.3. These facts, together with the analysis of the Bader charges (shown in next section), suggest that the insulating/metallic character is determined by the electron doping of the manganite through the polarity of the nickelate side (La$^{3+}$O$^{2-}$/Mn$^{3+}$O$^{4-}_2$). The presence of a single layer nickelate results in an insufficient polarity effect, a consequent charge localization and a strong hybridization of the occupied orbitals, as discussed above.

**Figure 5:**
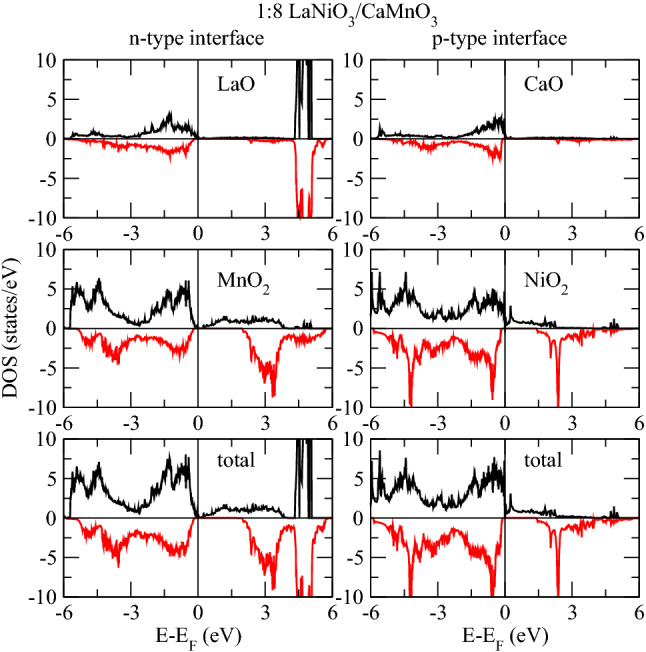
Interfacial layer densities of states of 1:8 at both interfaces.

**Figure 6:**
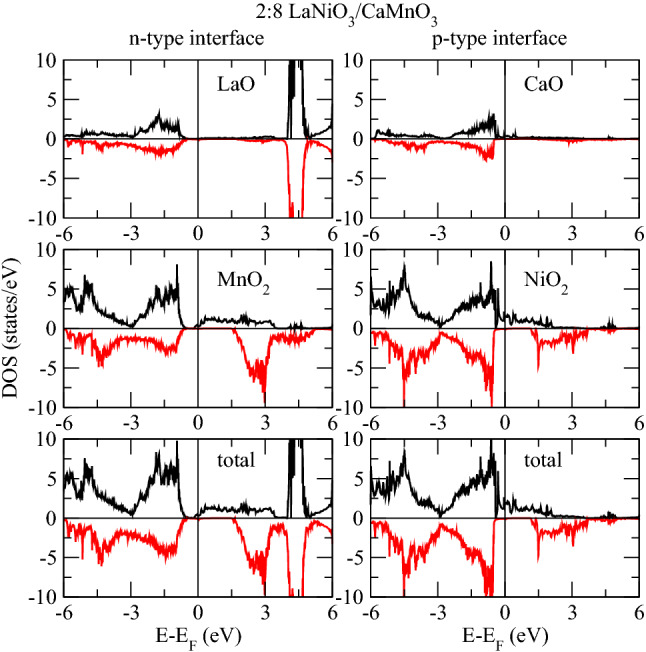
Interfacial layer densities of states of 2:8 at both interfaces.

**Figure 7:**
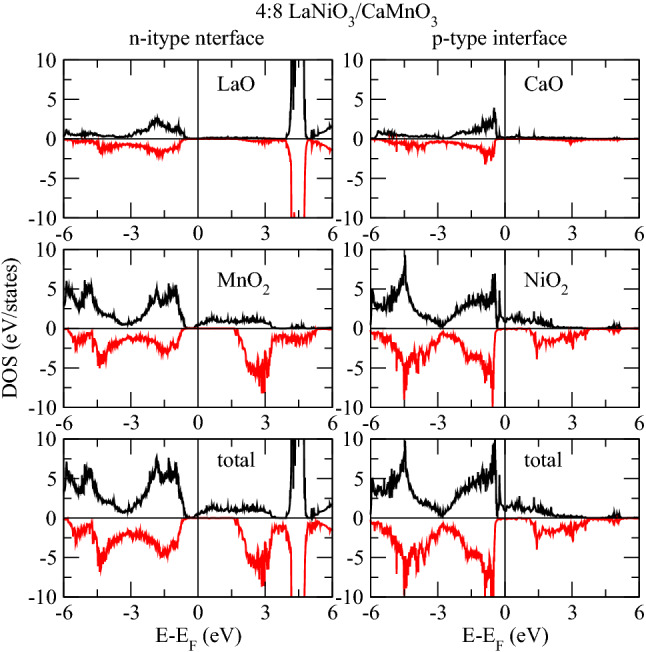
Interfacial layer densities of states of 4:8 at both interfaces.

The Mn $e_{g}$ orbitals in the 2:8 and 4:8 cases are downshifted, contributing to the metallicity of these superlattices. Moreover, there is a considerable overlap between the Mn and Ni $d_{3z^2-r^{2}}$ orbitals around E$_F$ across the *p*-IF, especially in the 2:8 case (compare Figs. 3 and 4) which indicates a Mn-Ni exchange interaction and which contributes to the orbital reconstruction. The interfacial layer-projected DOS was calculated by combining the total DOS of the all atoms in each layer (plotted in Figs. 5, 6 and 7). For metallic superlattices, the states at E$_F$ are attributed to NiO$_2$ and MnO$_2$ layers with prominent contribution from the NiO$_2$ layers at the *p*-IF. Note that the contributions from LaO and CaO layers are negligible. These combined features result in rich charge redistribution and the consequent interfacial magnetism which we discuss next.

Extremely localized appearance of LaNiO$_3$ (its thickness being ultrathin, as is in 1:8 superlattice), sandwiched between two bulk CaMnO$_3$ slabs results in the introduction of large local lattice distortion, hence sudden disorder effects^60^ (which is also manifested in prominently different octahedral distortion, as discussed before) and consequently localized electronic states, resembling quasi-2D character. Simultaneously, the presence of considerable electronic states in the vicinity of the E$_F$, suggests that the metallic-insulating transition cannot be merely due to Coulomb repulsion mechanism, but Anderson localization^61^ may be involved. Such phenomenon is widely known to occur in LaNiO$_3$ nanoparticles^62^ and films or superlattices^63, 64^. Most of Hamiltonian based studies consider the interaction between the $e_{g}$ orbitals^65, 66^.

On the other hand, the increase in LaNiO$_3$ thickness induces a higher electrostatic potential in the superlattice due to the polarity of the LaO and NiO$_2$ layers, therefore charge redistribution takes place to avoid the polar divergence^67^. This fills the $d_{x^{2}-y^{2}}$ at both interfaces and contributes to a reduced electronic localization, see the significant increase of DOS at the E$_F$ (in 2:8 and 4:8 DOS from Figs. 1, 3 and 4). The latest work^68^ also demonstrated the metallic behavior of LaNiO$_3$/LaAlO$_3$ thin films when LaNiO$_3$ thickness is reduced down to 2 unit cells. The 4:8 system shows clear electronic population at E$_F$ for both spin channels. Similar findings were reported experimentally in (LaNiO$_3$)$_n$/(SrMnO$_3$)$_2$ superlattice where the system transitions from insulator (n=1) to hopping conductor (n=2) to metal (n=4) with increasing LaNiO$_3$ thickness^69^. Our results are also in line with findings of dimensionality induced metal-insulator transition in LaNiO$_3$/LaAlO$_3$ superlattice^70^. Due to its large computational cost, no further increase in the LaNiO$_3$ thickness is considered, however, importantly, metallicity can be predicted according to the trend from our total DOS features. Next we analyse charge transfer of different layers to give insights into polarity of nicklate site as mentioned earlier.

### Charge transfer

Charge transfer analysis of each layer (two formula unit per layer) can shed light on the magnetic exchange mechanism between magnetic ions. From Bader charges of individual layers and comparing them to the values in the bulk components, we find that in the 1:8 system, the LaNiO$_3$ region loses 0.14e, while the adjacent CaMnO$_3$ region gains 0.04e and 0.06e at the *n*- and *p*-IF, respectively. This indicates that a charge transfer from the LaNiO$_3$ layer to the CaMnO$_3$ layer occurs. The charge redistribution in the 2:8 and 4:8 superlattices is different; in these cases electrons are gained at the *n*-IF by both manganite and nickelate; in the 2:8 (4:8) superlattice, the magnitude of the transferred charge across the *n*-IF is 0.27e (0.25e) and 0.04e (0.08e), into the LaNiO$_3$ and CaMnO$_3$ layers, respectively. Consistently, an electron depletion occurs at both sides of the *p*-IF, 0.22e (0.35e) and 0.11e (0.12e). The charge modification of individual interfacial magnetic atoms is lower than their average effective transferred charges, pointing to additional participation of O, Ca, and La atoms in the charge redistribution.

Notice that the charge gained by the Mn atoms is at most 0.02e throughout the superlattice with small variations. This indicates the existence of mixed valence state Mn$^{4+}$ and Mn$^{4-\delta }$($\delta$ < 0.02e). Ni atoms gain (lose) charges systematically at the *n*-IF(*p*-IF) and demonstrate mixed valence state of Ni$^{3\pm \delta }$($\delta$ < 0.04e). Note that the existence of mixed valence state was also advanced in a previous work^16^. This is also indicative of slightly different spin exchange processes in *n*-IF as compared to *p*-IF, which demands further investigation, and might have interesting consequences^71^.

Since in the experimental work^16^, the authors don’t discuss individual interfacial behavior, it is not straightforward to compare and correlate the amount of charge transfer with experiment. Although the charge transfer is small in our case, it is known that DFT underestimates charge localization; small charges are reported also elsewhere^72^. In general, polarity mismatch results in charge transfer in variety of nanostructures^73, 74^. Also it is important to note that LaNiO$_3$/CaMnO$_3$ superlattices in experiment are grown on polar LaAlO$_3$ single crystal substrate, which may have additional effects in the charge transfer and other effects. We observe a prominent overall charge transfer from *p*-IF region to *n*-IF region, mediated via the polar LaNiO$_3$ layers. With increase in LaNiO$_3$ thickness (for 2:8 and 4:8 cases), we observe more charge modification of interfacial CaMnO$_3$ layers as compared to inner layers.

### Magnetic properties

Dependence of electronic DOSs and charge transfers on LaNiO$_3$ thickness and interface type has important consequence on the magnetic properties of these systems. In order to understand superlattice magnetism, we study the magnetic behavior and spin ordering of the bulks. First we compare the strained bulk systems with their respective unstrained bulk counterpart to identify the effects of strain. Thereafter, we will discuss the results for superlattices in the light of what happens in the strained bulk components.

The effect of strain on the magnetic ordering of CaMnO$_3$ is small due to relatively smaller lattice mismatch. Since strained bulk CaMnO$_3$ at low temperature is known to demonstrate G-AFM ordering, $U_{eff}$= 3 eV is a reasonable choice for an adequate description of its electronic properties^47^. According to Kanamori’s rules^75^, in ligand-mediated flat bonds (when M-O-M is 180$^{\circ }$) super-exchange interaction gives rise to an AFM ordering between magnetic ions with same kind of *d*-shell, but results in a FM ordering between magnetic ions with more than half-filled *d*-shell and less than half-filled *d*-shell. Mn is in a Mn$^{4+}$ valence state which is also confirmed by the same charge state of strained CaMnO$_3$ from our analysis. This affirms that the AFM ordering in CaMnO$_3$ is originated from the super-exchange interaction between Mn$^{4+}$ ions mediated via O atoms, as widely known^76–78^; this is often observed in transition metal oxides^77, 78^. The average Mn magnetic moments is 2.9$\mu _B$ which is close to the experimental value of 2.84$\mu _B$^79^.

Now coming to bulk LaNiO$_3$ which demonstrates strong polar nature, as well as larger lattice mismatch with substrate, the strain has more prominent effects one electronic and magnetic behavior. Under strain, the magnetic moment of Ni atom changes to 0.37$\mu _B$(1.17$\mu _B$) from unstrained case of 0.02$\mu _B$(1.12$\mu _B$), when $U_{eff}$= 0($U_{eff}$= 4.3 eV). Depending on the possible types of bonds between the magnetic ions, there can be three different paths of exchange interactions in the superlattices i.e. Ni-Ni, Mn-Mn and Ni-Mn mediated through corner shared oxygen ligands. The different distances between magnetic ions and their angles connected to a ligand can influence the strength of exchange interaction since these factors directly affect the charge and spin distribution. Inter (and intra) spin exchange coupling due to Pauli principle combined with correlated nature of electrons as well as electron hopping (kinetic exchange) makes the spin exchange process more sophisticated^80^.

**Figure 8:**
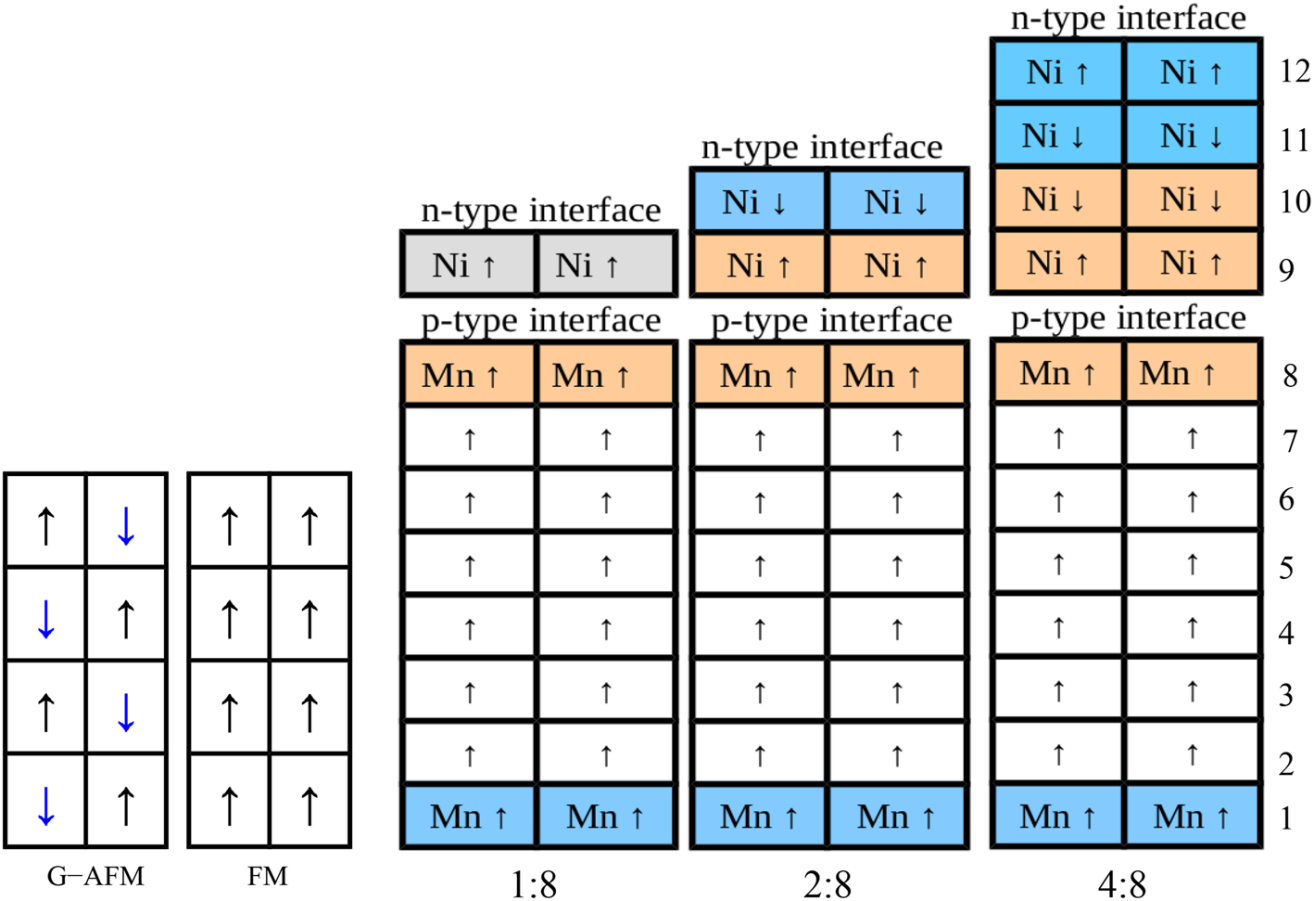
Schematic view of the spin orientations of bulk (G-AFM and FM CaMnO$_3$) and LaNiO$_3$/CaMnO$_3$ superlattices with varying LaNiO$_3$ thickness. Orange and blue colors depict the near *p* and *n* type interface regions respectively whereas the gray color in 1:8 case depicts the proximity to both interfaces due to single NiO$_2$ layer.

Schematic view of the spin orderings for the bulk and superlattices are shown in Fig.8 and interfacial magnetic moments are given in Table 1. We have tested FM and G-type AFM spin alignments (imposed on CaMnO$_3$) in these superlattices and found that FM aligned CaMnO$_3$ is energetically favourable in superlattices irrespective of the choice of $U_{eff}$ on Ni atoms [with the 91 meV, 103 meV, and 272 meV (when $U_{eff}^{Ni}$= 0) lower energies than AFM ordering energies in 1:8, 2:8 and 4:8 cases, respectively.] With increasing $U_{eff}$ on Ni, the trend remains same, demonstrating even higher stability of FM phase which is in agreement with experimental results^16^. In addition, regardless of the LaNiO$_3$ thickness, both LaNiO$_3$ and CaMnO$_3$ layers contribute to the superlattice magnetism in which the CaMnO$_3$ is FM aligned whereas LaNiO$_3$ is AFM aligned near both *n*- and *p*-IFs, see Fig.8. It is worth considering that strained bulk of a very similar compound, LaMnO$_3$ and SrMnO$_3$ show very similar behaviour^24^.

Experimental work^16^ confirms the existence of Ni magnetism in both insulating and metallic LaNiO$_3$/CaMnO$_3$ superlattices. They highlighted the importance of the polar compensation on magnetism and showed the existence of super-exchange (between Ni−Mn) and double-exchange interaction(between Mn−Mn) in these systems. Redistribution of charges and, orbital occupations in the LaNiO$_3$ layers from our results indicates towards the polar compensation and consequent existence of super-exchange interaction within Ni ions. Particularly in 1:8 case, the confinement effect as mentioned earlier plays a major role. In Ref.^35^, authors have also shown theoretically the dominant role of the confinement effects on the Ni magnetism.

Interfacial magnetic atoms (Mn and Ni) show FM alignments for all cases with the exception only in 2:8 case, *n*-IF, where AFM aligned ordering was observed (see Fig.3c, minority DOS of $d_{x^{2}-y^{2}}$ orbitals), resulting in average magnetic moments of −1.353$\mu _B$, and 3.01$\mu _B$ for Ni and Mn atoms, respectively. As compared to bulk magnetic moment, Ni moment increases by 0.02$\mu _B$ in 1:8 case. For 2:8 and 4:8 cases, Ni moment increases by 0.18$\mu _B$, 0.15$\mu _B$ at *n*-IF and decreases by 0.29$\mu _B$, 0.37$\mu _B$ at *p*-IF, respectively.

Note that all Mn atoms in the CaMnO$_3$ layers demonstrate FM ordering, in contrast to AFM bulk phase. Although this is mainly due to the strain induced by the formation of the superlattice, electronic effects can induce a modulation of spin magnitudes across the superlattice, Mn atoms have mixed valence states in all superlattices. Note that a double-exchange mechanism driven by mixed valency can support an antiferromagnetic order^81^, but here the strain is inducing a transition (compare refs.^82, 83^. This FM ordering in LaNiO$_3$/CaMnO$_3$ also correlates to the systematic displacement of the Mn ions, as discussed in Section III-A. Small O$^{2-}$ magnetic moments ranging from 0.01$\mu _B$ to 0.13$\mu _B$ as found in all superlattices arise from the strong hybridization of Mn and Ni atoms with O 2*p* states; this also indicates the possible charge transfer between O and Mn or Ni. O ions play an important role in interfacial magnetism, through its participation in all charge redistribution and spin exchanges. O vacancy^84^, doping^85^ etc are other known factors that can affect the magnetism. Additionally, specific substrate orientation can result in an improved interfacial magnetism and exchange bias effect^86^ as suggested by recent experiments. Note that our results for different LaNiO$_3$/CaMnO$_3$ superlattices show similar features as reported for LaNiO$_3$/LaMnO$_3$ superlattices in Ref.^54^, which suggests that reduced dimensionality and interface asymmetry in LaNiO$_3$/LaMnO$_3$ superlattices are main factors that lead to complex magnetic behaviour. The superlattice magnetism in our system can be explained by double-exchange FM interaction of CaMnO$_3$, super-exchange AFM within LaNiO$_3$ and super-exchange FM at the interface mediated by oxygen ligands.

## Conclusions

We presented the structural, electronic, and magnetic properties of epitaxially strained (LaNiO$_3$)$_n$/ (CaMnO$_3$)$_m$ superlattices with varying LaNiO$_3$ layer thickness. Metal-insulator transition is achieved with decreasing LaNiO$_3$ thickness from 4 unit cells to one unit cell, with a good agreement with what observed in^16^. The insulating character of the thinnest LaNiO$_3$ is attributed to the Anderson localization of electronic states, caused by disorder effects, also reflected in respective octahedral distortion. This elucidates uncovered details of the role of electronic orbitals across a nickelate/manganite interface; the possibility that orbital hybridization is combined with Anderson localization is hinted. Ni atoms demonstrate an important influence on the magnetism at the interface. The AFM coupling of the Ni atoms within all the modeled superlattices matches that observed in experiment^15^, suggesting possibilities for different magnetic behavior of the LaNiO$_3$ based heterostructures. The FM ordering of CaMnO$_3$ within the superlattices are correlated to the double-exchange interaction due to strain and the existence of mixed valence states. Moreover, the non monotonic evolution of the magnetic ground state from a thin and insulating nickelate layer to a large and half-metallic one, such as the thickness-dependent Ni-Mn exchange coupling, provides an additional path to engineering and tuning the interfacial magnetism in complex perovskite oxide heterostructures. Key factors for superlattice magnetism in our system are identified as double-exchange FM interaction of CaMnO$_3$, super-exchange AFM within LaNiO$_3$ and super-exchange FM at the interface mediated by oxygen ligands. As the functionalities increase with complexity, the present work has important implications for future functional device design.

## Data Availability

The datasets used and/or analysed during the current study available from the corresponding author on reasonable request.
